# Multicentre genetic diversity study of carbapenem-resistant Enterobacterales: predominance of untypeable pUVA-like *bla*_KPC_ bearing plasmids

**DOI:** 10.1093/jacamr/dlad061

**Published:** 2023-05-26

**Authors:** Patricia J Simner, Yehudit Bergman, Yunfan Fan, Emily B Jacobs, Srividya Ramakrishnan, Jennifer Lu, Shawna Lewis, Ann Hanlon, Pranita D Tamma, Michael C Schatz, Winston Timp, Karen C Carroll

**Affiliations:** Department of Pathology, Division of Medical Microbiology, Johns Hopkins University School of Medicine, 600 N Wolfe Street, Meyer B1-125, Baltimore, MD, USA; Department of Medicine, Division of Infectious Disease, Johns Hopkins University School of Medicine, Baltimore, MD, USA; Department of Pathology, Division of Medical Microbiology, Johns Hopkins University School of Medicine, 600 N Wolfe Street, Meyer B1-125, Baltimore, MD, USA; Department of Biomedical Engineering, Johns Hopkins University, Baltimore, MD, USA; Department of Pathology, Division of Medical Microbiology, Johns Hopkins University School of Medicine, 600 N Wolfe Street, Meyer B1-125, Baltimore, MD, USA; Department of Computer Science, Johns Hopkins University, Baltimore, MD, USA; Department of Biomedical Engineering, Johns Hopkins University, Baltimore, MD, USA; Center for Computations Biology, Whiting School of Engineering, Johns Hopkins University, Baltimore, MD, USA; Department of Pathology, Division of Medical Microbiology, Johns Hopkins University School of Medicine, 600 N Wolfe Street, Meyer B1-125, Baltimore, MD, USA; Department of Pathology, Division of Medical Microbiology, Johns Hopkins University School of Medicine, 600 N Wolfe Street, Meyer B1-125, Baltimore, MD, USA; Department of Pediatrics, Division of Pediatric Infectious Diseases, Johns Hopkins University School of Medicine, Baltimore, MD, USA; Department of Computer Science, Johns Hopkins University, Baltimore, MD, USA; Simons Center for Quantitative Biology, Cold Spring Harbor Laboratory, Cold Spring Harbor, NY, USA; Department of Biology, Johns Hopkins University, Baltimore, MD, USA; Department of Medicine, Division of Infectious Disease, Johns Hopkins University School of Medicine, Baltimore, MD, USA; Department of Biomedical Engineering, Johns Hopkins University, Baltimore, MD, USA; Center for Computations Biology, Whiting School of Engineering, Johns Hopkins University, Baltimore, MD, USA; Department of Pathology, Division of Medical Microbiology, Johns Hopkins University School of Medicine, 600 N Wolfe Street, Meyer B1-125, Baltimore, MD, USA

## Abstract

**Objectives:**

Carbapenem-resistant Enterobacterales (CRE) are an urgent public health threat. A better understanding of the molecular epidemiology and transmission dynamics of CRE is necessary to limit their dissemination within healthcare settings. We sought to investigate the mechanisms of resistance and spread of CRE within multiple hospitals in Maryland.

**Methods:**

From 2016 to 2018, all CRE were collected from any specimen source from The Johns Hopkins Medical Institutions. The isolates were further characterized using both phenotypic and genotypic approaches, including short- and/or long-read WGS.

**Results:**

From 2016 to 2018, 302 of 40 908 (0.7%) unique Enterobacterales isolates were identified as CRE. Of CRE, 142 (47%) were carbapenemase-producing CRE with KPC (80.3%) predominating among various genera. Significant genetic diversity was identified among all CRE with high-risk clones serving as major drivers of clonal clusters. Further, we found the predominance of pUVA-like plasmids, with a subset harbouring resistance genes to environmental cleaning agents, involved in intergenus dissemination of *bla*_KPC_ genes.

**Conclusions:**

Our findings provide valuable data to understand the transmission dynamics of all CRE within the greater Maryland region. These data can help guide targeted interventions to limit CRE transmission in healthcare facilities.

## Introduction

The CDC Antibiotic Resistance Threats in the United States, 2019 report highlights that antimicrobial resistance contributes to over 2.8 million infections and greater than 35 000 deaths annually in the USA.^1^ Of antimicrobial-resistant organisms, the CDC has assigned the highest threat level to carbapenem-resistant Enterobacterales (CRE), declaring they require urgent public health attention.^1,2^ A better understanding of the molecular epidemiology and transmission dynamics of CRE is necessary to limit their dissemination within healthcare settings.

There are two major mechanisms driving the spread of CRE in the USA: (i) vertical transmission of successful clones [e.g. ST258 *Klebsiella pneumoniae* carbapenemase (KPC)-producing *K. pneumoniae*] and (ii) horizontal spread of carbapenemase-producing CRE (CP-CRE) through promiscuous plasmids and/or associated mobile genetic elements (e.g. IncFII, pKpQIL, Tn*4401*).^3^ To date, a primary focus of public health efforts has been on CP-CRE because their carbapenemase genes can spread with great ease across various Gram-negative species or even between genera through transmissible genetic elements.^4^ However, we believe both mechanisms merit further investigation to understand their relative importance in contributing to the CRE epidemic. Moreover, studies of CRE transmission in the USA have generally been limited to outbreak settings or have focused on specific species or carbapenemase genes resulting in incomplete data for a comprehensive understanding of CRE transmission dynamics.^5,6^ Our objective was to understand the mechanisms of resistance and spread that contribute to the successful dissemination of CRE, regardless of species, encountered within hospital settings. These data are critical in guiding interventions to limit CRE transmission in acute care facilities.

## Material and methods

### Bacterial isolates

From 1 January 2016 to 31 December 2018, all ertapenem-not-susceptible (intermediate or resistant) Enterobacterales were collected from any specimen source from The Johns Hopkins Medical Institutions including, The Johns Hopkins Hospital (1162 beds), Bayview Medical Center (420 beds) and Howard County General Hospital (267 beds) in Maryland, USA. Bacterial genus and species were identified using MALDI-TOF MS (Bruker Daltonics Inc., Billerica, MA, USA). Carbapenem antimicrobial susceptibility testing (AST) results were determined clinically using the BD Phoenix Automated System (BD Diagnostics, Sparks, MD, USA) and interpreted following CLSI guidelines.^7^ The phenotypic modified carbapenem inactivation method (mCIM) was performed on all CRE isolates to differentiate CP-CRE from non-carbapenemase-producing CRE (non-CP-CRE).^7^ All CRE isolates were stored frozen using Microbank beads (Pro-Lab Diagnostics, Richmond Hill, ON, Canada) at −80°C until further testing was pursued. This study was approved by The Johns Hopkins University School of Medicine institutional review board.

### AST

Unique patient isolates (i.e. one isolate per patient per species) were subcultured from frozen stock along with a second subculture on 5% sheep blood agar prior to performing AST. Repeat AST was performed on isolates using lyophilized Sensititre broth microdilution (BMD) CTNX2F or GN7F and MDRGNX2F panels (Thermo Fisher Scientific, Waltham, MA, USA). Results were interpreted following CLSI guidelines with the exception of eravacycline, omadacycline, plazomicin and tigecycline, which were interpreted applying FDA susceptibility interpretive criteria.^8^ Quality control (QC) was set up on each day of testing.

### WGS

All unique patient isolates underwent WGS. WGS was performed by short-read Illumina sequencing (HiSeq and MiSeq); CP-CRE underwent additional sequencing using long-read nanopore sequencing. Genomic DNA was extracted from pure cultures using the DNeasy PowerSoil Pro Kit (Qiagen, Hilden, Germany). Short-read sequencing libraries were prepared using Nextera DNA Flex Library prep Kit and Nextera DNA CD Indexes (Illumina, San Diego, CA, USA). Paired-end reads were generated using a HiSeq rapid SBS kit, v2 (2 × 250 bp, 500 cycle kit) or Illumina MiSeq (2 × 300, 600 cycle kit). Long-read genomic sequencing was performed using the MinION or GridION (Oxford, England) sequencing instrument. Each nanopore sequencing library was prepared using 1 µg DNA with the 1D ligation kit (SQK-LSK108, Oxford Nanopore Technologies) and sequenced using R9.4.1 flowcells (FLO-MIN106). MinKNOW software was used to collect sequencing data. Sequencing data were basecalled using the ONT basecaller, Albacore v2.3.3. A subset of 200k reads passing the basecalling filter were then used to assemble genomes using canu v1.7.^9^ Assemblies were corrected with Illumina reads using Pilon v1.22 in conjunction with bowtie2 v2.3.3.1.^10,11^ Isolates sequenced on the Illumina platform were assembled using SPAdes (v3.14.1).^12^ Assembly statistics are summarized in Table S1 (available as Supplementary data at *JAC-AMR* Online). Assemblies were annotated with prokka (v1.14.6).^13^ The pangenome was constructed from annotated assemblies using roary default settings (v3.7.0), which also generates a core genome alignment.^14^ The core genome alignment was fed into RAxML (v8.2.12) to develop a phylogenetic tree using ‘-m GTRGAMMA’ with 100 bootstrap replicates.^15^ Plasmid incompatibility types were detected using PlasmidFinder.^16^ Whole genome assemblies were deposited to the sequence read archive (SRA) under bioproject PRJNA496461 and PRJNA686978.

### Plasmid assemblies

pUVA-like plasmids were assembled using Canu v 1.7. Draft assemblies were polished with Illumina short reads in response to iterative Pilon runs.^9,10^ Plasmids were circularized and rotated to match the origin of replication of the reference pUVA01 plasmid.^17,18^ Plasmids were annotated using Prokka.^13^ Multiple sequence alignments were generated using MAFFT, and phylogenetic trees were constructed using phyML to compute the sequence similarity of plasmids harbouring *bla*_KPC_.^19,20^ Additionally, a whole plasmid phylogenetic tree was constructed to visualize the sequence similarity of the plasmids. To identify structural variations in plasmid sequences with respect to the reference pUVA01 plasmid, full-length plasmid sequences were aligned to the pUVA01 reference using the nucmer utility (MUMmer3 package).^21^ Structural variants in the plasmid assemblies were detected and corrected (Assemblytics software).^22^ Variations found using nucmer and assemblytics were plotted using Circa (http://omgenomics.com/circa/), relative to the pUVA01 reference sequence.

### Comparison of pUVA-like plasmids

For comparison, 42 092 RefSeq plasmid sequences were downloaded and aligned to the reference pUVA01 plasmid using minimap2 and followed by Bowtie2.^10,19^ A phylogenetic tree was generated based on sequence similarity using Mash for the subset of RefSeq plasmids that aligned to pUVA01.^23^ The resulting RefSeq plasmids matching pUVA01 were compared against the ∼10 kb insertion from the KLPN_262 assembled plasmid using the nucmer utility to identify any plasmids with similar structural variants.^17^

## Results

### Bacterial isolates

From 2016 to 2018, 40 908 unique Enterobacterales isolates were recovered from patient specimens. Of these, 302 (0.7%) were carbapenem-resistant (Table S2). CRE were recovered from multiple sources, including urine (98, 32.5%); respiratory (68, 22.2%); rectal surveillance cultures (40, 13.2%); wound (38, 12.6%); blood (35, 11.6%); tissue (14, 4.6%); and sterile fluids (10, 3.3%).

Of CRE, 142 (47%) were CP-CRE and 150 (53.0%) were non-CP-CRE (Table 1). The most common CP-CRE were *Klebsiella pneumoniae* complex (77, 55.4%), *Enterobacter cloacae* complex (19, 13.3%), *Escherichia coli* (19, 13.3%) and *Klebsiella oxytoca* (11, 7.8%), whereas the most common non-CP-CRE were *E. cloacae* complex (61, 38.1%), *K. pneumoniae* complex (44, 27.5%), *E. coli* (26, 16.3%) and *Klebsiella aerogenes* (13, 8.1%).

**Table 1.** Distribution of carbapenem-resistant Enterobacterales from 2016 to 2018 at three hospitals within The Johns Hopkins Health System

	2016	2017	2018	Δ2016 to 2018 *P* value^a^	Total
All Enterobacterales	14 174	13 818	12 916		40 908
CRE	110 (0.8)	106 (0.8)	86 (0.7)		302 (0.7)
CP	48 (43.6)	59 (55.7)	35 (40.7)		142 (47.0)
Non-CP	62 (56.4)	47 (44.3)	51 (59.3)		160 (53.0)
*Klebsiella pneumoniae* complex	2369	2291	2153		6813
CR *K. pneumoniae* complex	48 (44.6% of CRE; 2.0% of total)	44 (41.5% of CRE; 1.9% of total)	29 (33.7% of CRE; 1.3% of total)	0.0015	121 (40.1% of CRE; 1.8% of total)
CP	30 (62.5)	32 (72.7)	15 (51.7)		77 (63.6)
Non-CP	18 (37.5)	12 (27.3)	14 (48.3)		44 (36.4)
*Enterobacter cloacae* complex	696	697	671		2064
CR *E. cloacae* complex	30 (27.8% of CRE; 4.3% of total)	28 (26.4% of CRE; 4.0% of total)	22 (25.6% of CRE; 3.3% of total)	0.32	80 (26.7% of CRE; 3.9% of total)
CP	5 (16.7)	7 (25.0)	7 (31.8)		19 (23.8)
Non-CP	25 (83.3)	21 (75.0)	15 (68.2)		61 (76.2)
*Escherichia coli*	7849	7633	6930		22, 412
CR *E. coli*	13 (11.8% of CRE; 0.2% of *E. coli*)	17 (16.0% of CRE; 0.2% of *E. coli*)	15 (17.4% of CRE; 0.2% of *E. coli*)	0.48	45 (14.9% of CRE; 0.2% of *E. coli*)
CP	4 (30.8)	9 (52.9)	6 (40.0)		19 (42.2)
Non-CP	9 (69.2)	8 (47.1)	9 (60.0)		26 (57.8)
*Klebsiella aerogenes*	232	249	258		739
CR *K. aerogenes*	3 (2.8% of CRE; 1.3% of total)	2 (1.9% of CRE; 0.8% of total)	9 (10.5% of CRE; 3.5% of total)	0.11	14 (4.7% of CRE; 1.9% of total)
CP	0	0	1 (11.1)		1 (7.1)
Non-CP	3 (100)	2 (100)	8 (88.9)		13 (92.9)
*Klebsiella oxytoca*	366	389	396		1151
CR *K. oxytoca*	1 (0.9% of CRE; 0.3% of total)	5 (4.7% of CRE; 1.3% of total)	5 (5.8% of CRE; 1.3% of total)	0.12	11 (3.7% of CRE; 1.0% of total)
CP	1 (100)	5 (100)	4 (80.0)		10 (90.9)
Non-CP	0	0	1 (20.0)		1 (9.1)
*Citrobacter freundii* complex	256	251	266		773
CR *C. freundii* complex	7 (6.5% of CRE; 2.7% of total)	2 (1.9% of CRE; 0.8% of total)	0	0.028	9 (3.0% of CRE; 1.2% of total)
CP	5 (71.4)	2 (100)	0		7 (77.8)
Non-CP	2 (28.6)	0	0		2 (22.2)
*Serratia marcescens*	370	340	337		1047
CR *S. marcescens*	4 (3.7% of CRE; 1.1% of total)	3 (2.8% of CRE; 0.9% of total)	1 (1.2% of CRE; 0.3% of total)	0.21	8 (2.7% of CRE; 0.8% of total)
CP	0	3 (100)	1 (100)		4 (50)
Non-CP	4 (100)	0	0		4 (50)
*Proteus mirabilis*	1087	1132	983		3202
CR *P. mirabilis*	1 (0.9% of CRE; 0.09% of total)	0	3 (3.5% of CRE; 0.3% of total)	0.27	4 (1.3% of CRE; 0.1% of total)
CP	0	0	0		0
Non-CP	1 (100)	0	3 (100)		4 (100)
*Morganella morganii*	198	177	209		589
CR *M. morganii*	0	2 (1.9% of CRE; 1.1% of total)	0	0.96	2 (0.7% of CRE; 0.3% of total)
CP	0	0	0		0
Non-CP	0	2 (100)	0		2 (100)
Other Enterobacterales	751	659	713		2123
CR	1^b^ (0.9% of CRE; 0.1% of total)	3^c^ (2.8% of CRE; 0.5% of total)	2^d^ (2.3% of CRE; 0.3% of total)	0.53	6 (2.0% of CRE; 0.3% of total)
CP	1 (100)	1	1 (50)		3 (50)
Non-CP	0	2	1 (50)		3 (50)

Values in parentheses are percentages. CP, carbapenemase-producing; CR, carbapenem-resistant; CRE, carbapenem-resistant Enterobacterales; non-CP, non-carbapenemase-producing.

a *P* value calculated by chi-square.

b 1 CP-*Pantoea* spp.

c CP-*Proteus vulgaris* group, non-CP *Providencia suartii*, non-CP *Hafnia alvei*.

d 1 non-CP *Citrobacter koseri*, 1 CP-*Providencia rettgeri*.

### Molecular epidemiology of CRE

Among CP-CRE, common carbapenemase genes identified were as follows: *bla*_KPC_ (114, 80.3%), *bla*_NDM_ (8, 5.6%), *bla*_OXA-48-like_ (4, 2.8%), and the combination of *bla*_NDM_ and *bla*_OXA-48-like_ (4, 2.8%; Table 2). Two *bla*_SME_ were detected in two of eight (25%) CR-*Serratia marcescens* isolates. Nine different genera harboured *bla*_KPC_, with *K. pneumoniae* complex being the most common. There was a decreasing trend in the number of CP-CRE harbouring *bla*_KPC_ from 2016 (41; 85%) to 2018 (26; 74%). The detection of *bla*_NDM_ and *bla*_OXA-48-like_ enzymes was limited to *E. coli* and *K. pneumoniae* complex, with the exception of a *bla*_NDM_- and *bla*_OXA-48-like_-producing *Providencia rettgeri.* The detection of *bla*_NDM_ was most common in *E. coli* (six of eight NDM producers; 75%), with *bla*_NDM-5_ (five of six *bla*_NDM_*E. coli* isolates) being the predominant genotype. In contrast, the two *K. pneumoniae* complex isolates harboured *bla*_NDM-1_. Isolates harbouring *bla*_OXA-48-like_ appeared in 2017 (five, 8.5% of CP-CRE) and persisted into 2018 (three, 8.6% of CP-CRE).

**Table 2.** Molecular epidemiology of carbapenemase-producing Enterobacterales from 2016 to 2018 from three facilities within the Johns Hopkins Health System

	2016	2017	2018	Total
**CRE**	110	106	86	302
**CP**	48 (43.6)	59 (55.7)	35 (40.7)	142 (47.0)
**KPC**	41 (85.4)	47 (79.7)	26 (74.3)	114 (80.3)
*Klebsiella pneumoniae* complex	28 (68.3)	28 (59.6)	12 (46.2)	68 (59.6)
*Enterobacter cloacae* complex	4 (9.8)	6 (12.8)	6 (23.1)	16 (14.0)
*Escherichia coli*	3 (7.3)	4 (8.5)	3 (11.5)	10 (8.8)
*Citrobacter freundii* complex	4 (9.8)	1 (2.1)		5 (4.4)
*Klebsiella oxytoca*	1 (2.4)	4 (8.5)	4 (15.4)	9 (7.9)
*Pantoea* spp.	1 (2.4)			1 (0.9)
*Serratia marcescens*		2 (4.3)		2 (1.8)
*Proteus vulgaris* group		1 (2.1)		1 (0.9)
*Klebsiella aerogenes*			1 (3.8)	1 (0.9)
**NDM**	5 (10.4)		3 (8.6)	8 (5.6)
*Escherichia coli*	3 (60.0)		3 (100)	6 (75.0)
*Klebsiella pneumoniae* complex	2 (40.0)			2 (25.0)
**OXA-48-like**		3 (5.1)	1 (2.9)	4 (2.8)
*Escherichia coli*		1 (33.3)	1 (100)	2 (50)
*Klebsiella pneumoniae* complex		2 (66.7)		2 (50)
**NDM and OXA-48-like**		2 (3.4)	2 (5.7)	4 (2.8)
*Klebsiella pneumoniae* complex		2 (100)	1 (50)	3 (75.0)
*Providencia rettgeri*			1 (50)	1 (25.0)
**Other**	2 (4.2)	7 (11.9)^a^	3 (8.6)^b^	12 (8.5)

Values in parentheses are percentages. CP, carbapenemase-producing; CRE, carbapenem-resistant Enterobacterales

a 1 SME-2-producing *S. marcescens.*

b 1 SME-3-producing *S. marcescens.*

Among non-CP-CRE known to have inducible, chromosomal *ampC* β-lactamase genes, *ampC* genes were the most common β-lactamase genes identified [e.g. *bla*_ACT_/*bla*_MIR_ among *E. cloacae* complex (54; 88.5%), *bla*_CMY_ among *Citrobacter freundii* complex (2; 100%) and *bla*_DHA_ among *Morganella morganii* (2; 100%)]. ESBL genes were the most common β-lactamase genes identified in Enterobacterales isolates not harbouring inducible *ampC* genes [e.g. *bla*_CTX-M_ among *K. pneumoniae* complex (22; 50%) and *E. coli* (14; 53.8%)]. A few notable exceptions were observed. *bla*_DHA_ was identified in five (11.4%) non-CP-CR *K. pneumoniae* complex, and *bla*_CMY_ was identified in three (11%) non-CP-CR *E. coli*. Finally, the ESBL genes *bla*_CTX-M_ or *bla*_SHV-12_ were identified in 8% (4% each) of *E. cloacae* complex isolates.

### AST profiles

Table 3 summarizes the AST profiles among CRE over the study period. The most active agents against CRE were polymyxin B (86.0%), colistin (87.2%), imipenem/relebactam (87.6%), amikacin (87.8%), cefiderocol (89.2%), meropenem/vaborbactam (92.4%), tigecycline (93.7%), plazomicin (94.4%) and ceftazidime/avibactam (94.8%). Non-CP-CRE were significantly more likely to be susceptible to aminoglycosides, aztreonam, ceftolozane/tazobactam, fluoroquinolones, tetracyclines and trimethoprim/sulfamethoxazole (*P* < 0.05).

**Table 3.** Antimicrobial susceptibility profiles among carbapenem-resistant Enterobacterales from 2016 to 2018

	All CRE					Non-CP-CRE					CP-CRE					*P* value
	MIC (mg/L)					MIC (mg/L)					MIC (mg/L)					
	*N*	Range	%S	%I	%R	*N*	Range	%S	%I	%R	*N*	Range	%S	%I	%R	
Amikacin	254	≤4 to >32	87.8	4.3	7.9	121	≤4 to >32	97.5	0.8	1.7	133	≤4 to >32	78.9	7.5	13.5	<0.00001
Ampicillin	151	≤8 to >16	0.7	0.0	99.3	83	≤8 to >16	1.2	0.0	98.8	68	≤8 to >16	0.0	0.0	100.0	
Ampicillin/sulbactam	151	≤4/2 to >16/8	1.3	1.3	97.4	83	≤4/2 to >16/8	2.4	0.0	97.6	68	≤4/2 to >16/8	0.0	2.9	97.1	
Aztreonam	253	≤1 to >16	4.3	2.8	92.9	121	≤1 to >16	8.3	1.6	90.1	132	≤1 to >16	0.8	3.8	95.4	0.003
Cefazolin	151	≤1 to >16	0.0	0.0	100.0	83	≤1 to >16	0.0	0.0	100.0	68	2 to >16	0.0	0.0	100.0	
Cefepime	254	≤2 to >16	15.3	27.2 (SDD)	57.5	121	≤2 to >16	15.7	33.1 (SDD)	51.2	133	≤2 to >16	15.0	21.8 (SDD)	63.2	0.0008
Cefiderocol	251	≤0.03 to >32	89.2	6.8	4.0	118	≤0.03 to >32	88.1	8.5	3.4	133	≤0.03 to >32	90.2	5.3	4.5	0.6
Cefotaxime	107	≤1 to >32	0.9	3.8	95.3	38	≤1 to >32	2.6	7.9	89.5	69	2 to >32	0.0	1.4	98.6	
Ceftazidime	253	≤1 to >16	6.7	2.0	91.3	121	≤1 to >16	6.6	1.7	91.7	132	≤1 to >16	6.8	2.3	90.9	0.95
Ceftazidime/avibactam	251	≤0.12/4 to >32/4	94.8	N/A	5.2	118	≤0.12/4 to >32/4	98.3	N/A	1.7	133	≤0.12/4 to >32/4	91.7	N/A	8.3	0.2
Ceftolozane/tazobactam	251	≤0.06/4 to >8/4	10.4	11.1	78.5	118	≤0.06/4 to >8/4	15.2	13.6	71.2	133	0.25/4 to >8/4	6.0	9.0	85.0	0.02
Ceftriaxone	151	≤0.5 to >32	4.0	2.0	94.0	83	≤0.5 to >32	4.8	2.4	92.8	68	≤0.5 to >32	2.9	1.5	95.6	0.6
Ciprofloxacin	254	≤0.25 to >2	30.3	5.5	64.2	121	≤0.25 to >2	48.8	5.8	45.4	133	≤0.25 to >2	13.5	5.3	81.2	<0.00001
Colistin	227	≤0.25 to >4	N/A	87.2	12.8	117	≤0.25 to >4	N/A	91.5	8.5	110	≤0.25 to >4	N/A	82.7	17.3	
Delafloxacin	251	≤0.12 to >1	28.3	5.2	66.5	118	≤0.12 to >1	43.2	9.3	47.5	133	≤0.12 to >1	15.0	1.5	83.5	<0.00001
Doripenem	254	≤0.12 to >4	41.0	16.1	42.9	121	≤0.12 to >4	62.0	16.5	21.5	133	≤0.12 to >4	21.8	15.8	62.4	<0.00001
Doxycycline	107	≤2 to >16	43.0	14.0	43.0	38	≤2 to >16	28.9	13.2	57.9	69	≤2 to >16	50.7	14.5	34.8	0.03
Eravacycline	251	0.06–8	70.1	N/A	29.9 (NS)	118	0.06–8	63.6	N/A	36.4 (NS)	133	0.06–8	75.9	N/A	24.1 (NS)	0.03
Ertapenem	254	≤0.25 to >8	0.0	11.4	88.6	121	≤0.25 to >8	0.0	18.2	81.8	133	≤0.25 to >8	0.0	5.3	94.7	
Gentamicin	253	≤1 to >8	68.0	3.5	28.5	121	≤1 to >8	85.1	0.0	14.9	132	≤1 to >8	52.3	6.8	40.9	<0.00001
Imipenem	254	≤2 to >16	32.7	15.0	52.3	121	≤2 to >16	62.8	13.2	24.0	133	≤2 to >16	5.3	16.5	78.2	<0.00001
Imipenem/relebactam	251	≤0.03/4 to >16/4	87.6	4.8	7.6	118	≤0.03/4 to >16/4	91.5	5.1	3.4	133	0.06/4 to >16/4	84.2	4.5	11.3	0.08
Levofloxacin	254	≤0.5 to >8	35.4	4.3	60.3	121	≤0.5 to >8	52.1	3.3	44.6	133	≤0.5 to >8	20.3	5.3	74.4	<0.00001
Meropenem	254	≤0.25 to >8	33.8	15.0	51.2	121	≤0.25 to >8	57.0	11.6	31.4	133	≤0.25 to >8	12.8	18.0	69.2	<0.00001
Meropenem/vaborbactam	251	≤0.008/8 to >16/8	92.4	2.0	5.6	118	≤0.008/8 to >16/8	94.9	3.4	1.7	133	0.015/8 to >16/8	90.2	0.8	9.0	0.2
Minocycline	254	≤1 to >16	54.3	15.0	30.7	121	≤1 to >16	49.6	13.2	37.2	133	≤1 to >16	58.7	16.5	24.8	0.1
Nitrofurantoin	151	≤32 to >64	43.7	27.8	28.5	83	≤32 to >64	37.4	33.7	28.9	68	≤32 to >64	51.5	20.6	27.9	0.08
Omadacycline	251	0.5 to >8	69.3	12.0	18.7	118	0.5 to >8	63.5	10.2	26.3	133	0.5 to >8	74.5	13.5	12.0	0.06
Piperacillin/tazobactam	254	≤8/4 to >64/4	5.1	12.6	82.3	121	≤8/4 to >64/4	7.4	18.2	74.4	133	≤8/4 to >64/4	3.0	7.5	89.5	0.1
Plazomycin	251	≤0.12 to >4	94.4	0.8	4.8	118	≤0.12 to >4	98.2	0.9	0.9	133	≤0.12 to >4	91.0	0.7	8.3	0.01
Polymyxin	107	≤0.25 to >4	N/A	86.0	14.0	38	≤0.25 to >4	N/A	86.8	13.2	69	≤0.25 to >4	N/A	85.5	14.5	
Tetracycline	151	≤4 to >8	58.3	11.9	29.8	83	≤4 to >8	54.2	35.0	10.8	68	≤4 to >8	63.3	13.2	23.5	0.3
Ticarcillin/clavulanic acid	107	≤16 to >128	0.0	0.9	99.1	38	≤16 to >128	0.0	2.6	97.4	69	>128	0.0	0.0	100.0	
Tigecycline	254	≤0.25 to >8	93.7	4.3	2.0	121	≤0.25 to >8	89.3	7.4	3.3	133	≤0.25 to >8	97.7	1.5	0.8	0.005
Tobramycin	254	≤1 to >8	53.9	7.9	38.2	121	≤1 to >8	77.7	3.3	19.0	121	≤1 to >8	32.3	12.0	55.7	<0.00001
Trimethoprim/sulfamethoxazole	253	≤0.5/9.5 to >4/76	44.3	N/A	55.7	121	≤0.5/9.5 to >4/76	58.7	N/A	41.3	132	≤0.5/9.5 to >4/76	31.1	N/A	68.9	<0.00001

I, intermediate; N/A, not applicable; NS, non susceptible; R, resistant; S, susceptible; SDD, susceptible dose-dependent; MIC, minimum inhibitry concentration; CP, carbapenemase-producing; CR, carbapenem-resistant; CRE, carbapenem-resistant Enterobacterales; non-CP, non-carbapenemase-producing.

### Phylogeny among CRE

**Figure 1.**
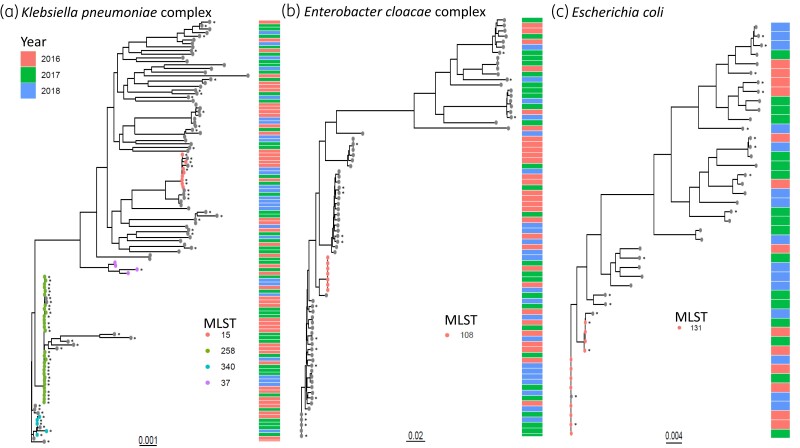
Phylogenetic analysis of the top three carbapenem-resistant Enterobacterales from a 2016 to 2018 cohort from three facilities within the Johns Hopkins Health System. Number of substitutions per site; STs are listed if greater than three isolates were identified belonging to the ST. Asterisk denotes carbapenemase-producing carbapenem-resistant Enterobacterales isolates.

Focusing on the top three CRE encountered, significant heterogeneity was identified among bacterial STs (Figure 1). The predominant ST among CR-*K. pneumoniae* complex was ST258 (32, 26.4%) with the majority of ST258 isolates harbouring *bla*_KPC_ [*bla*_KPC-2_ (17) or *bla*_KPC-3_ (13)]. Outside of ST258, a few additional clusters of STs were identified across *K. pneumoniae* complex isolates. Three (2.5%) CR-*K. pneumoniae* complex isolates—all of which were non-CP-CRE—belonged to ST37. Additionally, six (5.0%) KPC-3-producing *K. pneumoniae* complex belonged to ST340. A small cluster (*n* = 5) of KPC-2-producing ST15 *K. pneumoniae* complex isolates was identified in 2016 and persisted over the next 2 years. Overall, STs were diverse, with 31 different STs identified among non-CP and 22 STs among CP-*K. pneumoniae* complex (Figure 1a). SNP analysis revealed between 0 and <1000 SNPs identified among the core genome of *K. pneumoniae* complex isolates.

Significant ST diversity was identified among CR-*E. cloacae* complex isolates, with 29 STs among non-CP and 13 STs among CP isolates (Figure 1b). Two small clusters of three isolates and two isolates of CP-*E. cloacae* complex ST93 and ST316, respectively, were identified. The predominant ST amongst non-CP-CRE *E. cloacae* complex isolates was ST108 (seven, 11.5%), with small clusters of two isolates each of ST32, ST66, ST113, ST533 and ST807. Isolates differed between 0 and <139  000 SNPs among the core genome of *E. cloacae* complex isolates.

Among CR-*E. coli*, ST131 was the most common ST among the CP-CRE (five, 23.8%; all *bla*_KPC-3_) and non-CP-CRE isolates (eight, 33.3%). Outside of ST131, STs were diverse, with 13 and 16 different STs identified among CP-CRE and non-CP-CRE, respectively (Figure 1c). *E. coli* core genomes varied between isolates from 0 to  < 24  000 SNPs.

### Plasmid analysis

A total of 38 different plasmid incompatibility types were identified across the 302 CRE isolates. On average, CP-CRE contained six plasmid incompatibility types (range: 0–10) compared with non-CP-CRE, which averaged three plasmid incompatibility types (range: 0–9). Common plasmid types were as follows: IncFII (170, 13.4%), IncFIB (169, 13.3%), ColRNAI (133, 10.5%), IncFIA (60, 4.7%), IncR (58, %), RepA (50, 3.9%), IncN (37, 2.9%) and IncX (37, 2.9%). Fifty CRE (16.5%) contained an untypeable RepA plasmid, of which 48 (96%) harboured *bla*_KPC_. Other associations included IncF plasmids assigned to multiple incompatibility types (e.g. IncFII/InFIB/IncHI1B/IncR) harbouring *bla*_KPC_ among ST258 and ST340 *K. pneumoniae* complex, IncR plasmids harbouring *bla*_KPC-2_ among ST258 *K. pneumoniae* complex, and ColKP3 plasmids harbouring *bla*_OXA-181_ or *bla*_OXA-232_ (five of the eight OXA-48-like producers).

### Untypeable RepA plasmid analysis

**Figure 2.**
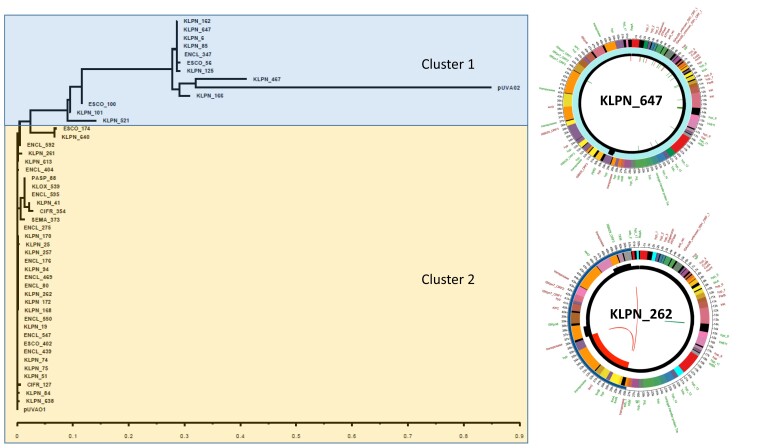
Phylogenetic tree of pUVA-like plasmids identified among carbapenem-resistant Enterobacterales showing number of substitutions per site. KLPN_647: Cluster 1 representative untypeable RepA plasmid isolated from a *Klebsiella pneumoniae* isolate. Note the deletion observed in the *virB4* gene, which was present among all plasmids in this cluster. KLPN_262: Cluster 2 representative untypeable RepA plasmid isolated from a *K. pneumoniae* complex isolate. Note the 10 kb insertion containing the *bin3* gene and *frmRAB* operon. Outer track is the gene identification with gene boundaries highlighted in multiple colours, blue line is the Tn*4401* element found in Cluster 2 plasmids (at 28–51k), the black track highlights the read coverage at the position, and the inner track highlights structural variations compared with pUVA01. Structural variants are colour coded as red for insertion, green for deletion and pink for repeat expansion. The green arcs are the deletion track and the red arcs are the insertion track.

The predominance of *bla*_KPC_ carriage by the untypeable RepA plasmid led to further investigations of this plasmid. The untypeable RepA plasmid, present in 43.9% of KPC producers, shared a similar backbone to pUVA01, plasmids that caused an intergenus CRE outbreak of KPC at a facility in Virginia, but still demonstrated variability with multiple recombination, insertion and deletion events.^18^ In our cohort, the untypeable RepA plasmids clustered into two groups based on phylogenetic similarity (Figure 2).

The first group of 12 untypeable RepA plasmids varied from 40 to 83 kb in size and lacked the Tn*4401* element and were distantly related to the pUVA02 plasmid (Figure 2, KLPN_647 representative plasmid). They harboured *bla*_KPC-3_ and were isolated from nine *K. pneumoniae* complex of various STs (ST11, ST15, ST16, ST273, ST340), two *E. coli* and a single *E. cloacae* complex. A number of single base insertions and deletions occurred throughout the untypeable RepA plasmids, compared with pUVA01. These mutations generally occurred in the region of plasmid replication and maintenance genes. For all plasmids in this cluster, a deletion was observed in the *virB4* gene, which encodes an inner membrane ATPase that is part of the type IV secretion system responsible for conjugative transfer.

The second, larger cluster of untypeable RepA plasmids closely resembled the pUVA01 plasmid and included 34 plasmids ranging in size from 40 to 57 kb (Figure 2; KLPN_262 representative plasmid). These plasmids harboured *bla*_KPC-3_ (24, 71%) or *bla*_KPC-2_ (10, 29%). Organisms within this cluster of plasmids were identified in more diverse species than the first cluster and included *K. pneumoniae* complex (17), *E. cloacae* complex (10), *E. coli* (2), *C. freundii* complex (2), *Pantoea* species (1), *K. oxytoca* (1) and *S. marcescens* (1). Interestingly, 13 of the 34 plasmids (38%) acquired a 7–10 kb insertion that contained *bin3* (recombinase), *frmA*, *frmB* and/or *frmR* genes. *frm* genes are associated with aldehyde resistance and likely contribute to plasmid survival in the hospital environment where disinfectants containing aldehydes or chlorine are commonly used.

**Figure 3.**
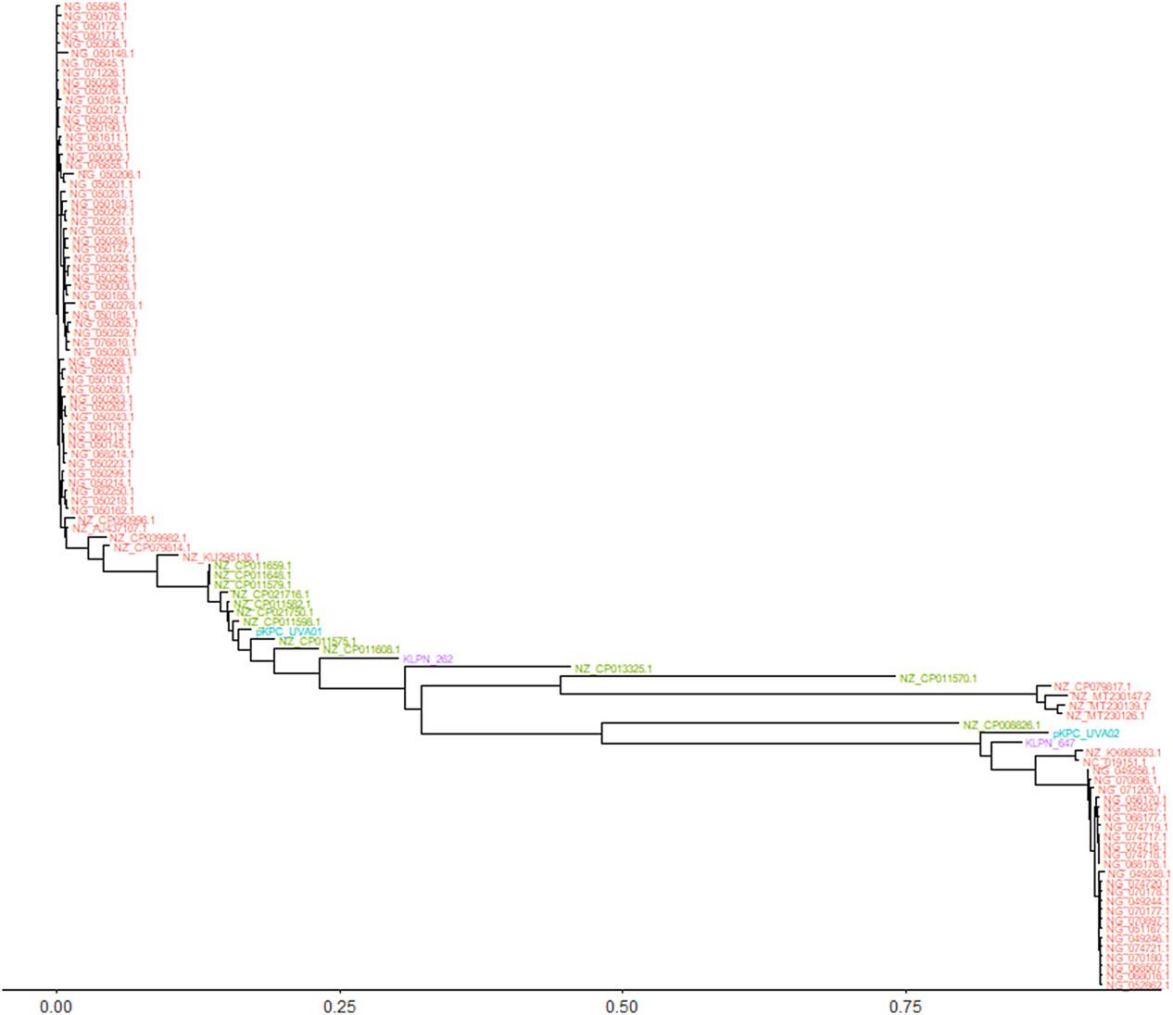
Phylogenetic tree demonstrating the genome distances as calculated by Mash^23^ between the 104 RefSeq plasmids that align to the reference pUVA01 plasmid, the representative assembled KLPN_262 and KLPN_647 (purple), and the reference pUVA01 and pUVA02 (blue). Highlighted in green is the subset of 14 plasmids that contain the same ∼10 kb insertion identified in the KLPN_262 cluster of assembled plasmids.

Of 42 092 RefSeq plasmids, 5001 plasmids successfully aligned against the reference pUVA01 plasmid using minimap2. The more sensitive alignment using Bowtie2 yielded only 104 RefSeq plasmids matching the reference pUVA01. When compared with the assembled representative KLPN_262 plasmid using nucmer, 14 of these RefSeq sequences contained the same ∼10 kb insertion. Figure 3 shows the phylogenetic tree for the 104 RefSeq plasmids matching the pUVA01 reference plasmid, alongside pUVA01, pUVA02, KLPN_262 and KLPN_647.

## Discussion

Our findings demonstrate high genetic diversity among CRE and further define the successful establishment of a promiscuous *bla*_KPC_-bearing plasmid among various Enterobacterales within the hospital setting. Interestingly, a subset of the *bla*_KPC_-bearing pUVA-like plasmids demonstrated a 10 kb insert encoding resistance genes to environmental cleaning agents. These data can inform effective approaches to limit CRE transmission in healthcare settings, including infection prevention and antibiotic stewardship strategies.

Overall, the proportion of unique Enterobacterales identified in our cohort was stable and remained at <1% throughout the 3 year study period, consistent with data from the CDC indicating stabilization of CRE rates from 2013 to 2017.^1^ The plateauing of CRE prevalence is likely a reflection of successful, targeted infection prevention efforts implemented at both local and national levels to curb their spread. As an example, the CDC has advocated screening for CP-CRE colonization upon hospital admission for patients who had overnight stays in healthcare facilities outside the USA in the prior 6 months.^4^ Similarly, they have recommended communicating to other facilities a patient’s CP-CRE status during transitions of care to limit their spread across the healthcare continuum.

*K. pneumoniae* complex has been implicated as the Enterobacterales species with the greatest contribution to the CRE epidemic. Interestingly, we found a significant decrease in the proportion of *K. pneumoniae* complex isolates identified as CR over the study period. Our data are in agreement with those of Wilson *et al.*,^24^ who reported decreasing proportions of CR-*K. pneumoniae* complex across US Veterans Health Administration facilities from 2006 to 2015. They also reported an increase or a ‘second epidemic’ wave of CR-*E. cloacae* complex isolates originating from the West Coast and progressing eastward across the USA. The proportion of CR-*E. cloacae* complex was stable in our cohort, but it is yet unclear if this so-called ‘second epidemic’ will impact the mid-Atlantic region.

We observed an even split of carbapenem resistance mediated by CP and non-CP mechanisms across our cohort. This is similar to previous reports from our group from 2013 to 2016.^25,26^ This distribution varies by organism, with *K. oxytoca*, *C. freundii* complex and *K. pneumoniae* complex most commonly identified as CP-CRE, whereas *P. mirabilis*, *M. morganii*, *K. aerogenes* and *E. cloacae* complex were more likely to be non-CP-CRE. Clinical microbiology laboratories are encouraged to generate similar regional data to inform prioritization of carbapenemase detection in their facilities. Further, implementation of modalities with rapid turnaround times (e.g. same-day detection of carbapenemase production by phenotypic or molecular methods) should be considered for Enterobacterales most likely to be CP-CRE, to limit local dissemination and aid in patient management.

High genetic diversity was observed among all genera of CRE indicating independent mutational events through antibiotic selection pressure, clonal spread and mobile genetic elements all playing a role in the dissemination and molecular evolutionary trajectory of CRE. We observed the establishment of successful clones among our CRE including ST131 *E. coli* and ST258 *K. pneumoniae* complex among both non-CP-CRE and KPC-producing isolates. In contrast to a recent report highlighting the emergence of the *K. pneumoniae* complex ST307 clone in Texas, we identified only a single isolate belonging to this ST throughout the period of our cohort.^27^ The introduction of KPC-3 among ST131 *E. coli* raises concerns because of its international success and the global dominance of CTX-M-15 among ST131, resulting in considerable morbidity and mortality.^28^ Interestingly, ST108 *E. cloacae* complex, a clone previously associated with ESBL production, predominated in our acute care facilities.^29^ The ST108 cluster in our facilities were non-CP-CRE and did not produce ESBLs. Another US report found that ST171 was the predominant CR *E. cloacae* complex clone, underscoring important institutional differences in established clones conferring carbapenem resistance.^5^

In addition to clonal spread of successful CP-CRE clones, independent acquisition events of *bla*_KPC_ were notable and linked to an untypeable RepA plasmid bearing *bla*_KPC_.^30^ Our findings differ from reports from the UK noting the importance of an IncFII pKpQIL-like plasmid as well as IncR, ColRNAI and IncX3 plasmids contributing to the spread of *bla*_KPC_. Although we identified similar associations with IncF and IncR plasmids and *bla*_KPC_, the untypeable RepA plasmid predominated. The untypeable RepA plasmid identified in our cohort is closely related to the pKPC-UVA01/02 plasmids reported to cause an outbreak of KPC among isolates in four genera of Enterobacterales in Virginia, a US state adjacent to Maryland.^17,18^ Similarly, we identified two separate clusters of diverse pUVA-like plasmids among nine different genera associated with and without the transposition element Tn*4401*. Unlike previous reports, we identified the pKPC-UVA01-like plasmids among successful high-risk clones (ST258 and ST131).^18^ The combination of a broad host range and highly mobile plasmids among dominant clones is concerning. Furthermore, we demonstrated the evolution of the pUVA-like plasmids, even within a defined 1 month outbreak that we previously reported on, with mutations occurring in conjugative transfer genes.^31^

An interesting finding in our cohort was the detection of a 7–10 kb segment of the pUVA-01-like plasmids that harbours a recombinase gene (*bin3*) and *frmRAB* operon. The *frm* genes encode enzymes that function to detoxify a variety of compounds, such as *S*-formylglutathione hydrolase (*frmB*) and *S*-(hydroxymethyl) glutathione dehydrogenase (*frmA*), which are involved in resistance to and detoxification of formaldehyde and chlorine.^32–34^ The acquisition of these genes may contribute to the survival of isolates harbouring pUVA-01-like plasmids in the hospital environment where disinfectants containing aldehydes or chlorine are frequently used. Additionally, the use of these agents may also select for further horizontal transmission of *bla*_KPC_ among diverse genera. CRE survive in the hospital environment despite stringent environmental cleaning and disinfection.^31,35^ This is concerning because there are persistent reservoirs in the hospital setting for these pathogens to thrive, including sink drains in bathrooms, where environmental cleaning may lead to further selection, evolution and persistence—furthering intergenus spread and success in the hospital environment.^36^ Novel methods for cleaning and irradiation of organisms harbouring successful plasmids should be a focus of future interventions.

Our study has limitations. First, inclusion of isolates was limited to the greater Maryland region of the USA and may not be generalizable to other regions. However, our study is unique in including diverse hospitals in terms of bed size and patient population whereas current CRE epidemiology studies generally focus on large academic centres. Second, application of a hybrid approach including short- and long-read genome assemblies among CP-CRE isolates to study plasmid dynamics limits our understanding of the evolution of plasmids to those harbouring carbapenemase genes. Our findings underscore the importance of understanding the evolution of plasmids with and without resistance genes. Future studies should focus on analysing plasmids in all CRE. Similarly, a recent study highlighted the modular exchange that occurs among plasmids that harbour resistance genes and those that do not.^30^ Third, the acquisition of aldehyde and chlorine resistance genes identified among the untypeable RepA plasmids requires further investigation to understand the contribution to environmental persistence. Last, despite passing QC, an alert was sent out to customers of the Sensititer MDRGNX2F panels that the panels utilized in this study may produce lower cefiderocol MICs for some Gram-negative organisms.

Our findings provide valuable data to understand the transmission dynamics of all CRE within the greater Maryland region. Further, we demonstrate the predominance of *bla*_KPC_ pUVA-like plasmids with a subset harbouring resistance to environmental cleaning agents, potentially contributing to their persistence and dissemination in the healthcare setting. These data are significant to guide infection prevention efforts to reduce the development and sustainability of CRE within healthcare facilities.

## Supplementary Material

dlad061_Supplementary_Data

## Transparency declarations

P.J.S. reports grants and personal fees from Accelerate Diagnostics, OpGen Inc., bioMérieux, Inc. and BD Diagnostics; grants from Affinity Biosensors, Qiagen and Hardy Diagnostics; and personal fees from Shionogi, Inc., GeneCapture, outside the submitted work. W.T. has two patents (US Patent 8,748,091; US Patent 8,394,584) licensed to Oxford Nanopore Technologies. All other authors: none to declare.

## Supplementary data

Tables S1 and S2 are available as Supplementary data at *JAC-AMR* Online.
